# Dynamic balance and instrumented gait variables are independent predictors of falls following stroke

**DOI:** 10.1186/s12984-018-0478-4

**Published:** 2019-01-07

**Authors:** Kelly Bower, Shamala Thilarajah, Yong-Hao Pua, Gavin Williams, Dawn Tan, Benjamin Mentiplay, Linda Denehy, Ross Clark

**Affiliations:** 1 Department of Physiotherapy, The University of Melbourne, Parkville, VIC 3010 Australia; 2 School of Health and Sport Sciences, University of the Sunshine Coast, Queensland Sippy Downs, 4556 Australia; 3 Department of Physiotherapy, Singapore General Hospital, Bukit Merah, Singapore, 169608 Singapore; 4 Department of Physiotherapy, Epworth HealthCare, Richmond, VIC 3121 Australia; 5 La Trobe Sport and Exercise Medicine Research Centre, La Trobe University, Bundoora, VIC 3086 Australia; 6 Victorian Infant Brain Studies, Murdoch Children’s Research Institute, Parkville, VIC 3052 Australia

**Keywords:** Stroke, Outcome assessment, Postural balance, Gait, Accidental falls

## Abstract

**Background:**

Falls are common following stroke and are frequently related to deficits in balance and mobility. This study aimed to investigate the predictive strength of gait and balance variables for evaluating post-stroke falls risk over 12 months following rehabilitation discharge.

**Methods:**

A prospective cohort study was undertaken in inpatient rehabilitation centres based in Australia and Singapore. A consecutive sample of 81 individuals (mean age 63 years; median 24 days post stroke) were assessed within one week prior to discharge. In addition to comfortable gait speed over six metres (6mWT), a depth-sensing camera (Kinect) was used to obtain fast-paced gait speed, stride length, cadence, step width, step length asymmetry, gait speed variability, and mediolateral and vertical pelvic displacement. Balance variables were the step test, timed up and go (TUG), dual-task TUG, and Wii Balance Board-derived centre of pressure velocity during static standing. Falls data were collected using monthly calendars.

**Results:**

Over 12 months, 28% of individuals fell at least once. The faller group had increased TUG time and reduced stride length, gait speed variability, mediolateral and vertical pelvic displacement, and step test scores (*P* < 0.001–0.048). Significant predictors, when adjusted for country, prior falls and assistance (i.e., physical assistance and/or gait aid use) were stride length, step length asymmetry, mediolateral pelvic displacement, step test and TUG scores (*P* < 0.040; IQR-odds ratio(OR) = 1.37–7.85). With comfortable gait speed as an additional covariate, to determine the additive benefit over standard clinical assessment, only mediolateral pelvic displacement, TUG and step test scores remained significant (*P* = 0.001–0.018; IQR-OR = 5.28–10.29).

**Conclusions:**

Reduced displacement of the pelvis in the mediolateral direction during walking was the strongest predictor of post-stroke falls compared with other gait variables. Dynamic balance measures, such as the TUG and step test, may better predict falls than gait speed or static balance measures.

**Electronic supplementary material:**

The online version of this article (10.1186/s12984-018-0478-4) contains supplementary material, which is available to authorized users.

## Background

Stroke can result in a range of impairments which predispose an individual to falling. The incidence of community-based falls within the first six months following stroke is 37–73% [1–3], and the rate of falls in chronic stroke is approximately double that of healthy controls [4, 5]. People who have had a stroke are at a high risk of falls-related fractures [6]. Further adverse consequences may include fear of falling and subsequent reduced activity, deconditioning, and greater falls risk [7]. A recent systematic review identified mobility and balance variables (i.e., gait speed, timed up and go (TUG) and Berg Balance Scale) as the strongest predictors of falls after stroke [8]. Other significant factors included medications, mood, cognition and prior history of falls.

Gait speed (e.g., 10-m walk) is a common assessment for examining falls risk following stroke [9, 10], but it is possible that measures of movement quality during walking may assist risk assessment. Indeed, research has found that single limb support time asymmetry was an independent and strong predictor of falls after inpatient rehabilitation discharge [11]. Measures of step variability and “smoothness” during gait were also shown to be more strongly predictive of falls post stroke than other commonly-used clinical measures [12]. However, these studies were limited to a six-month follow-up period or were comprised of a small (*n* = 40) chronic stroke cohort [12]. Larger mediolateral pelvic displacement during walking has been demonstrated in people following stroke compared with healthy controls [13, 14]. While this variable has not previously been investigated in relation to falls risk after stroke, smaller mediolateral trunk displacement has been found in older adults with no falls history [15] or of the pelvis in those with worse balance [16]. Conversely, larger pelvic displacement was found to be predictive of falls in Parkinson’s disease [17].

There is currently no single balance test shown to be a superior predictor of falls following stroke. The Berg Balance Scale is a frequently used assessment for identifying post-stroke falls risk [1, 9]. Nonetheless, this test includes multiple items examining different aspects of balance performance and does not reveal which of these factors are more strongly reflective of risk [18]. Indeed, the Berg Balance Scale has also demonstrated poor falls prediction after stroke and is recommended for use in combination with other measures [19]. The TUG has shown to be predictive of falls after stroke [9], while the dual-task TUG has demonstrated superior predictive ability to the standard test in Parkinson’s disease [20]. Research in elderly cohorts has demonstrated variable findings, with a significant difference between fallers and non-fallers only for the standard TUG [21], equivalence between the two tests [22], and significance for falls prediction only for the cognitive dual-task TUG [23]. Prior research has also supported the use of the step test to predict post-stroke falls following inpatient discharge [1]. Static standing postural sway using a force platform has shown to differentiate post-stroke fallers [11, 24] or predict falls [25, 26], with findings tending to favour mediolateral variables [11, 26]. However, prior research has typically not included a comparison between a range of clinical and instrumented measures of balance and mobility to compare their relative strength for falls prediction. For example, studies have included only two clinical balance tests [1, 9] or only instrumented variables [26].

The current study aimed to comprehensively examine multiple aspects of gait and balance in relation to prospective falls after stroke over a 12-month period following inpatient rehabilitation discharge. Specifically, we aimed to identify which aspects of gait and balance were strongly associated with falls and whether instrumented variables, derived from using relatively accessible technologies, could add value to the standard clinical tests.

## Methods

### Participants

Individuals with stroke were consecutively recruited from inpatient rehabilitation facilities within Australia (two facilities; *n* = 30) and Singapore (two facilities; *n* = 66). Eligibility criteria were: 1) stroke occurring less than three months prior; 2) ability to walk 10 m independently or with minimal assistance; 3) adequate cognition and language to provide consent and participate in testing; 4) medically stable; and 5) no other condition that could confound physical testing (e.g., severe arthritis or progressive neurological disorder). No formal neuropsychological assessment of capacity was performed, but the treating team was consulted to determine whether potential participants had adequate cognition and communication. The study received ethical approval from the relevant institutions at each site. All participants provided written informed consent and language interpreters were used in Singapore as required.

### Procedures

Baseline testing occurred within one week prior to inpatient rehabilitation discharge. Participants were assessed by an experienced physiotherapist or exercise physiologist. Demographic and stroke characteristics (i.e., country, age, sex, time since stroke, lesion side and type of stroke) were collected in addition to the outcome measures described below. Baseline data included the Functional Independence Measure [27] (18–126, higher scores indicate better function), Modified Rankin Scale [28] (0–6; higher scores indicate less disability), Montreal Cognitive Assessment [29] (0–30; higher scores indicate better performance), Short Falls Efficacy Scale – International [30] (7–28; higher scores indicate less balance confidence), Hospital Anxiety and Depression Scale [31] (0–21; higher scores indicate greater anxiety and/or depression), presence of inattention (Star Cancellation Test) [32], comorbidities (Functional Comorbidity Index) [33] (0–18; higher scores indicate more comorbidities) and falls history.

### Gait variables

Individuals completed a stopwatch-timed 6 m walk test (6mWT) [34] at a comfortable pace, over a 10 m track. A Kinect camera (Microsoft, Redmond, USA) was used to obtain gait variables during a fast walk starting 6 m away from the camera. The data from one full stride occurring between approximately 1.8–4.0 m away from the camera was used for analysis, as this was within the middle capture volume of the Kinect where the most accurate data can be obtained. Spatiotemporal gait variables collected using the skeleton-tracking algorithm from the Kinect have demonstrated validity when compared to three-dimensional motion analysis systems in healthy adults and people following stroke [35–37]. Kinect-derived gait variables were: 1) gait speed: average anterior velocity of the central hip joint landmark, m/s; 2) stride length: the summed distance between ankle joint landmarks for two consecutive steps, m; 3) step width: the average distance between ankle joint landmarks in stance, m; 4) gait speed variability: SD of the anterior velocity, m/s; 5) step length asymmetry: maximum divided by minimum score, where 1 is perfect symmetry and larger scores represent greater asymmetry to either the left or right side; 6) mediolateral pelvic displacement: range between the furthest left and right positions of the central hip joint landmark, cm; and 7) vertical pelvic displacement: range between the lowest and highest vertical position of the central hip joint landmark, cm. Better performance is indicated by a faster gait speed and longer stride length; and typically by smaller values for step width, asymmetry, variability and pelvic movement. Shoes, gait aids and/or minimal physical assistance were used if needed for participant safety during the walking trials. The average of two successful trials was used for analysis.

### Balance variables

The TUG was used to assess dynamic balance [38] performed at both a comfortable pace and with a dual task component (i.e., counting backwards from a selected number between 60 and 100 in threes) [22]. The step test also assessed dynamic standing balance by asking participants to tap their foot on and off a 7.5 cm step for 15 s with either the more stroke affected or less affected limb in the stance position [39]. Static standing balance was assessed with a Wii Balance Board (WBB; Nintendo, Kyoto, Japan). This device has demonstrated excellent concurrent validity for static balance assessment in healthy and clinical populations when compared with other force platforms [40] and high test-retest reliability in people following stroke [41]. Individuals were asked to “stand as still as possible” for a duration of 30 s. Outcome variables included total, mediolateral and anteroposterior centre of pressure (COP) velocity and were determined using the analysis techniques contained in SeeSway, an online calculator incorporating Matlab and LabVIEW software [42]. Smaller values for the TUG and WBB tests indicate better performance, whereas higher step test scores indicate better performance. Shoes, gait aids and/or minimal physical assistance were used if needed for participant safety during the TUG trials. Nil aids or assistance was used for the step test or static balance trials. The average of two successful trials was used for analysis for all balance variables, with the exception of the step test, where participants performed several practice steps and then one trial as excellent reliability has been demonstrated using individual trials [39, 43].

The Kinect and WBBs were connected to a laptop running custom-written software created by author RAC (LabVIEW, National Instruments, USA). The WBBs were calibrated prior to data collection using a technique described previously. [44]

### Falls follow up

Participants prospectively recorded any falls over 12 months following discharge. A fall was defined as “an unexpected event in which the participants come to rest on the ground, floor or lower level” [45]. A 12-month calendar was provided for daily recording of falls, which were returned via mail each month [45]. Participants also provided written details of any fall including time, location, activity, and any injuries sustained. Telephone interviews were used to rectify missing data and confirm details of falls.

### Sample size

The effective sample size for this cohort was 51, based on the primary outcome of the number of falls modelled using multivariable ordinal regression [46]. Given the guideline of at least 10 patients per degree of freedom, a multivariable model can be reliably fitted if it has a complexity of ≤ five degrees of freedom.

### Statistical analysis

Descriptive statistics were presented for all baseline characteristics and outcome variables. Depending on the variable type and distribution, between-group differences for non-fallers and fallers (≥1 fall) were assessed using independent t-tests, Mann-Whitney *U* tests or Chi-Square tests. Stride length, step width and step length asymmetry data were removed from analysis for those using a walker (i.e., 2 or 4-wheeled walking frame; *n* = 4), due to the likely inaccuracy of the Kinect-based tracking of the lower limbs during these tests.

Each of the gait and balance variables were included separately as independent variables in an ordinal regression analyses, with prospectively collected falls as the dependent variable, adjusting for country, falls prior to stroke and assistance (i.e., minimal physical assistance and/or gait aid use). These variables were selected a priori as they were seen to be potential confounders (i.e., country and assistance) or previously shown to be strongly predictive of falls (i.e., prior falls) [2]. To avoid model overfitting, we included 3–4 covariates in the regression model. Therefore, a set of 19 regression analyses were performed with four independent variables included in each. Variables found to have a significant positive skew were log-transformed prior to analysis to reduce the influence of extreme predictor variables.

To estimate the utility of the gait and balance variables in providing additional information beyond a standard stopwatch-derived measure of gait speed, a second set of 17 regression analyses were performed using the 6mWT as an additional covariate (i.e., five independent variables in each). Missing 6mWT data for one participant was singly imputed using the covariates of fast gait speed, country and sex. A single regression imputation was performed as the missing data rate was small and unlikely to be missing completely at random [46, 47]. To further examine the potential influence of assistance on gait variables, two additional sets of regression analyses were performed with removal of all individuals requiring assistance or gait aids (i.e., 19 and 17 analyses with three and four independent variables in each).

Odds ratios and corresponding 95% confidence intervals were scaled to the IQR of each predictor to allow for a more clinically meaningful comparison between different variables which were quantified on different scales [46]. For variables which had an inverse relationship with falls, IQR-ORs were presented by comparing the 25th to 75th percentiles, thereby ensuring an OR ≥ 1.0 to facilitate comparison. Data were analysed using SPSS V23 and significance set at *P* < 0.05.

## Results

Of 96 individuals recruited, 81 (*n* = 25 Australia, *n* = 56 Singapore) completed baseline testing and 12 months prospective falls follow up. The reasons for loss of follow-up were voluntary withdrawal (*n* = 7), unable to contact or move overseas (*n* = 5), and death or further serious medical event (*n* = 3). There were no significant differences between those lost to follow-up and those retained in terms of baseline characteristics or clinical tests (6mWT, TUG and step test; *P* > 0.05). Participant characteristics are presented in Table 1. Over 12 months post discharge, 23/81 (28%) individuals fell at least once and 13/81 (16%) fell more than once. Significant differences between fallers and non-fallers were observed for the Modified Rankin Scale, Short Falls Efficacy Scale – International, prior stroke and falls in the 12 months preceding stroke (*P* = 0.008–0.046).

**Table 1:** Participant baseline characteristics and between-group differences

	All participants	Non-faller	Faller		
	(*n* = 81)	(*n* = 58)	(*n* = 23)	Test	*P*-value
Demographics					
Country (Singapore)	56 (69.1%)	42 (72.4%)	14:9 (60.9%)	3	0.310
Age	62.99 ± 13.22	62.83 ± 12.29	63.39 ± 15.63	1	0.864
Sex (male)	43 (53.1%)	31 (53.4%)	12 (52.2%)	3	0.917
Stroke details					
Time since stroke (days)	24.0 (20.0–34.5)	23.0 (20.0–33.3)	24.0(22.0–36.0)	2	0.515
Side of stroke lesion (right)	39 (48.1%)	27 (46.6%)	12 (52.2%)	3	0.771
Type of stroke (infarct)	64 (79.0%)	46 (79.3%)	18 (78.3%)	3	0.917
Functional measures, comorbidities and falls history					
Modified Rankin Scale (0–6)	2.83 ± 0.89	2.67 ± 0.91	3.22 ± 0.74	2	0.013*
FIM (18–126)	105.0 (93.0–116.5)	105.5 (94.0–118.0)	103.0 (92.0–115.0)	2	0.753
MoCA (0–30) (*n* = 78)	25.0 (23.0–28.0)	25.0 (23.0–27.0)	26.0 (20.8–28.0)	2	0.902
Inattention, star cancellation test < 44 (yes) (*n* = 80)	15 (18.8%)	11 (19.3%)	4 (17.4%)	3	0.843
HADS (0–21) (*n* = 77)	7.0 (3.0–12.0)	7.0 (3.0–12.5)	7.0 (3.0–11.5)	2	0.977
Short FES-I (7–28)	10.0 (8.0–15.0)	9.5 (7.0–14.0)	13.0 (10.0–19.0)	2	0.013*
Gait aid use (yes)	19 (23.5%)	11 (19.0%)	8 (34.8%)	3	0.130
Assistance for gait (yes)	16 (19.8%)	10 (20.8%)	6 (26.1%)	3	0.367
FCI (0–18)	1.60 ± 0.74	1.52 ± 0.71	1.83 ± 0.78	2	0.064
Prior stroke	5 (6.2%)	1 (1.7%)	4 (17.4%)	3	0.008*
Inpatient falls (yes)	15 (18.5%)	8 (13.8%)	7 (30.4%)	3	0.082
≥1 fall in 12 months preceding stroke (yes)	19 (23.5%)	10 (17.2%)	9 (39.1%)	3	0.046*

*Denotes a significance at P < 0.05

*Abbreviations, FCI Functional Comorbidity Index, FES-I Falls Efficacy Scale – International, FIM Functional Independence Measure, HADS Hospital Anxiety and Depression Scale, MoCA Montreal Cognitive Assessment*

*Note: Data presented as mean ± SD, median (IQR) or N (%), Test 1 independent t-test, Test 2 Mann-Whitney U test, Test 3 Chi-Square test*

### Falls details

Two participants each reported over 30 falls; one always when standing up from a chair and holding on to a walking frame, and the other always during community walking or climbing up or down stairs. No medical attention was sought for these falls. Apart from these two participants and missing details for 11/45 falls, 47% of falls occurred inside the home, 32% were related to going up or down stairs, and 24% occurred during walking. Six participants (7/42 falls) sought medical attention post fall but only one resulted in hospital admission for a shoulder fracture.

### Gait variables

Neither comfortable (stopwatch-derived) or fast (Kinect-derived) gait speed was significantly different between the faller and non-faller groups (Table 2). The faller group demonstrated significantly smaller stride length, gait speed variability, and mediolateral and vertical pelvic displacement. After adjusting for country, prior falls and assistance, significant predictors of falls were mediolateral pelvic displacement (IQR-OR = 7.85), stride length (IQR-OR = 4.23) and step length asymmetry (IQR-OR = 1.37). In regression models that also included the 6mWT as a covariate, only mediolateral pelvic displacement remained significant, with one IQR reduction in displacement (i.e., 5.38 cm versus 7.22 cm) indicating 6.75 times greater odds of falling.

**Table 2:** Between-group differences and adjusted regression analyses

	Non-fallers (*n* = 58)	Fallers (*n* = 23)	Between-group^a^	25th – 75th quartiles	Regression adjusted for country, prior falls and assistance		Regression adjusted for country, prior falls, assistance and 6mWT	
	Mean ± SD	Mean ± SD	*P*-value		IQR-OR (95% CI)	*P*-value	IQR-OR (95% CI)	*P*-value
*Gait variables*								
6mWT-comfortable, m/s (*n* = 57:23)	0.78 ± 0.36	0.61 ± 0.32	0.075	0.45–0.98	2.10 (0.90–4.89)^b^	0.086	N/A	N/A
Kinect-fast walk, m/s (*n* = 56:23)	0.93 ± 0.38	0.76 ± 0.37	0.106	0.57–1.16	1.89 (0.78–4.58)^b^	0.157	N/A	N/A
Stride length, m (*n* = 54:20)	1.03 ± 0.26	0.87 ± 0.31	0.043*	0.76-1.22	4.23 (1.14–15.66)^b^	0.031*	1.97 (0.26–14.79)^b^	0.511
Cadence, steps/min (*n* = 54:20)	107.84 ± 23.50	95.48 ± 31.61	0.114	91.41–122.08	2.48 (0.96–6.41)^b^	0.060	1.46 (0.45–4.81)^b^	0.530
Step width, m (*n* = 54:21)	0.15 ± 0.04	0.15 ± 0.04	0.943	0.12–0.17	1.19 (0.56–2.52)^b^	0.649	1.43 (0.65–3.13)^b^	0.376
Step length asymmetry, ratio (*n* = 53:20)^c^	1.15 ± 0.16	1.54 ± 1.10	0.458	1.06–1.16	1.37 (1.01–1.85)	0.040*	1.28 (0.96–1.69)	0.090
Gait speed variability, m/s (*n* = 56:23)^c^	0.17 ± 0.09	0.13 ± 0.04	0.048*	0.11-0.18	1.82 (0.78–4.26)^b^	0.169	1.24 (0.46–3.37)^b^	0.670
ML pelvic displacement, cm (n = 55:23)	7.00 ± 1.26	5.24 ± 1.39	< 0.001*	5.38-7.22	7.85 (2.56–24.05)^b^	< 0.001*	6.75 (2.20–20.74)^b^	0.001*
Vertical pelvic displacement, cm (*n* = 55:23)	3.42 ± 1.25	2.83 ± 0.96	0.047*	2.26-4.10	2.03 (0.74–5.56)^b^	0.168	1.25 (0.36–4.31)^b^	0.724
Balance variables								
TUG - normal, s (*n* = 58:23)^c^	16.49 ± 9.26	27.61 ± 23.84	0.014*	11.30-22.56	4.06 (1.44–11.46)	0.008*	7.84 (1.42–43.26)	0.018*
TUG - dual task, s (*n* = 54:19)^c^	21.47 ± 14.60	24.22 ± 3.06	0.148	11.91–25.44	1.30 (0.50–3.41)	0.589	1.38 (0.35–5.39)^b^	0.647
Step test - affected, taps/15 s (*n* = 58:23)	10.19 ± 5.08	6.35 ± 4.20	0.005*	6.00-12.50	4.50 (1.64–12.35)^b^	0.004*	5.28 (1.46–19.16)^b^	0.011*
Step test - less affected, taps/15 s (*n* = 58:23)	8.95 ± 4.81	4.74 ± 3.44	< 0.001*	4.50-10.00	6.98 (2.06–23.61)^b^	0.002*	10.29 (2.26–46.84)^b^	0.003*
COP vel EO total, cm/s (*n* = 57:22)^c^	1.52 ± 0.65	1.96 ± 1.13	0.186	1.14–1.36	1.26 (0.61–2.61)	0.527	1.29 (0.61–2.76)	0.505
COP vel EO ML, cm/s (*n* = 57:22)^c^	0.68 ± 0.35	0.83 ± 0.50	0.193	0.43–0.86	1.40 (0.70–2.80)	0.345	1.31 (0.64–2.69)	0.458
COP vel EO AP, cm/s (*n* = 57:22)^c^	1.20 ± 0.55	1.59 ± 1.00	0.128	0.89–1.40	1.18 (0.64–2.17)	0.590	1.24 (0.66–2.34)	0.508
COP vel EC total, cm/s (*n* = 56:22)^c^	2.11 ± 0.94	2.75 ± 1.64	0.172	1.45–2.86	1.17 (0.49–2.78)	0.725	1.20 (0.49–2.93)	0.687
COP vel EC ML, cm/s (*n* = 56:22)^c^	0.79 ± 0.37	0.99 ± 0.53	0.127	0.52–1.10	1.63 (0.60–4.44)	0.338	1.48 (0.53–4.14)	0.461
COP vel EC AP, cm/s (*n* = 56:22)^c^	1.78 ± 0.84	2.35 ± 1.54	0.230	1.21–2.32	1.06 (0.49–2.32)	0.882	1.12 (0.50–2.50)	0.788

*Denotes a significance at P < 0.05

^a^ *Between-group differences assessed using Mann-Whitney U tests;* ^*b*^ *an inverse relationship with falls exists, therefore IQR-OR compares the odds of having more falls among individuals with predictor values at the 25th percentile with the odds of having more falls among individuals with predictor values at the 75th percentile;* ^*c*^ *log-transformed for regression analysis due to significant positive skew*

*Abbreviations, 6mWT 6-m walk test, AP anteroposterior, COP centre of pressure, EC eyes closed, EO eyes open, IQR interquartile range, ML mediolateral, OR odds ratio, TUG timed up and go*

When all participants requiring physical assistance or gait aids (*n* = 24) were excluded from analysis, between-group differences were significant for stride length, gait speed variability, mediolateral and vertical pelvic displacement (Additional File 1). However, mediolateral pelvic displacement was the only significant gait variable for both regression models (IQR-OR = 9.35 and 8.54). Of note, mediolateral pelvic displacement had no significant correlation (Spearman’s rho) with gait speed, cadence or step width, for the whole sample (*n* = 74; absolute rho = 0.066–0.155; *P* = 0.191–0.574) or when those requiring aids or assistance were removed (*n* = 54; absolute rho = 0.083–0.225; *P* = 0.102–0.577).

### Balance variables

Fallers demonstrated significantly worse TUG and step test scores (Table 2). The same variables were significant following regression analysis adjusted for country, prior falls and assistance, and when the 6mWT was added as a covariate. One IQR increase in TUG scores indicated between 4.06–7.84 times greater falls risk. One IQR decrease in step test scores was associated with increased odds of falling of between 4.06–10.29.

## Discussion

The range of gait variables assessed in the current study revealed that a reduction in mediolateral pelvic displacement during fast-paced walking was the strongest predictor of falling following discharge from inpatient stroke rehabilitation. Mediolateral pelvic displacement was superior to, and independent of, a commonly used clinical measure of gait speed for predicting falls. However, mediolateral pelvic displacement is currently not easily and accurately quantifiable in clinical practice due to the technology required for assessment. The step test and TUG were more strongly predictive of falls than static balance variables or the standard measure of gait speed.

The faller group demonstrated smaller mediolateral pelvic amplitudes than the non-faller group which more closely approximated normal values (i.e., between 4 and 5 cm) [48]. Conversely, research has indicated greater lateral pelvic displacement in people following stroke compared with healthy controls [13, 14] and moderate strength negative correlations with gait speed [13, 49]. The smaller displacement of the pelvis in the frontal plane during walking in the faller group may reflect a compensatory or cautious movement strategy where the centre of mass is kept well within the boundary of the base of support to minimise lateral forces and increase stability. This is supported by research demonstrating reduced weight transfer to the paretic limb during walking in people with stroke [49, 50]. Difficulty in controlling lateral stability has been identified as a major contributor to falls in older adults [51] and research has shown smaller mediolateral trunk displacement in elderly individuals with a falls history [15].

Walking speed, stepping pattern or use of aids may have influenced movement at the pelvis. Interestingly, in the current study step width was not different between the faller groups and mediolateral pelvic displacement had no correlation with gait speed, cadence or step width. Although individuals needing aids or assistance were not excluded from testing in the current study, reduced mediolateral pelvic displacement was a significant predictor of falls despite assistance being included as a covariate or when these individuals were removed from the analyses. Indeed, prior research in a chronic stroke cohort has shown no significant effect of gait aid use on lateral pelvic displacement during walking [49]. Research suggests gait speed may increase with the use of aids [52] but others have found minimal impact on velocity, cadence or step symmetry [53, 54]. A study involving healthy adults demonstrated relatively low reliability and accuracy for Kinect-derived mediolateral pelvic displacement [36]. Measurement error in this variable could have led to over- or underestimation of the odd ratios [55]. Therefore, caution must be used when interpreting the findings of the current study. Research with a larger cohort is necessary to further explore the relationship between mediolateral pelvic displacement and falls, and the potential efficacy of training approaches targeting lateral weight transference to reduce falls risk.

In contrast to prior research [8], comfortable gait speed was not a significant predictor of falls. However, Harris et al. (2005) found that neither slow or fast gait speed measures discriminated between fallers and non-fallers in a chronic stroke cohort [56] and similar findings were seen for community-dwelling older adults [57]. Despite the faller group having slower gait speeds and a fast-paced speed which was similar to the comfortable speed of the non-faller group, the findings were not statistically significant. Significant predictive strength for stride length was demonstrated and this easily-assessed outcome may be superior to gait speed for evaluating falls risk. Step length asymmetry also warrants further investigation as this was found to be predictive of falls, though not independent of gait speed.

Dynamic balance assessments were better predictors of falls than were static measures. In contrast to the current study, a large study in older adults found no additive value of the TUG over gait speed for predicting falls [58]. However, this prior study involved a higher functioning cohort. The non-significant results for the dual-task TUG in the current study may have also been influenced by missing data from those unable or refusing to perform the test (*n* = 8). These participants were likely to have worse performance and their inclusion may have led to more significant findings. Although the use of aids or assistance was accounted for as a covariate in the regression analyses and remained significant with those individuals removed, research has suggested gait aid use is associated with improved performance of the TUG [59].

Previous research has similarly supported the use of the step test for falls prediction following inpatient stroke rehabilitation [1]. This test involves effective lateral weight transference onto the affected limb when it is in the stance position, and adequate clearance when tapping. This easy-to-implement test is therefore recommended as an important inclusion in the clinical assessment of falls risk post stroke.

None of the static balance COP velocity variables were significantly associated with falls. Static balance tasks are less reflective of activities where falls occur, such as during transfers and walking. Nonetheless, prior research has shown significant differences between fallers and non-fallers for mediolateral velocity SD and total COP sway area [11] and between non-fallers and repeat fallers for mediolateral and anteroposterior COP velocity [24]. While it is difficult to compare COP outcomes with these studies due to differences in equipment and analyses, the former cohort had slower gait speeds than the current study [11] and the latter was a chronic stroke sample. Another study investigating post-stroke falls following inpatient rehabilitation discharge, found root-mean-square COP variables to not be predictive of falls (versus no falls), but the mediolateral variable was significant for predicting increased fall rates when covariates were not controlled for [25]. Research in older adults has also suggested force platform measures of lateral control have predictive strength for falls [60].

The rate of falls in the current study (i.e., 28%) was lower than that previously reported in the literature [61]. This may be due to the inclusion of more highly functioning individuals who were able to walk with no more than minimal assistance, attending inpatient rehabilitation and discharged home.

There were some limitations associated with the methodology adopted in our study. Although participants were assessed at different time points post stroke, all were within the subacute window of recovery (i.e., less than three months post stroke) and assessment prior to discharge was selected as a clinically relevant timepoint for evaluating future falls risk. The findings may also have been influenced by loss of data for some participants mainly due to inability to complete the tests or technical issues. However, these represented a small proportion (typically < 5%) of the total participant numbers. The Kinect has a relatively small capture field of between 1.8–4.0 m from the camera. It would be useful to employ technologies which provided data over a larger number of steps and examine gait variables derived from comfortable-paced walking, as faster walking may result in more normal values for some gait variables [62]. Further, the Kinect was unable to accurately collect aspects of gait including temporal step measures [63]. Nonetheless, the depth-sensing technology used by the Kinect and other similar devices is recommended as a relatively accessible means of obtaining more detailed information on walking performance in clinical settings [64].

## Conclusions

Mediolateral pelvic displacement was found to be more strongly predictive of falls than other gait variables and was independent of a standard measure of gait speed. This variable has the potential to be assessed at relatively low-cost and using existing technologies. Stride length and step length asymmetry were also significant indicators of falls risk after stroke but were not independent of walking speed. Dynamic balance measures (i.e., the TUG and step test) were more strongly predictive of falls than static balance variables.

## Additional File

Additional File 1: (.pdf) Between-group differences and regression analyses with removal of those requiring physical assistance and/or gait aids (*n* = 57) (PDF 77 kb)

## Abbreviations

6mWT: six-metre walk test
COP: centre of pressure
FIM: Functional Independence Measure
TUG: timed up and go
WBB: Wii Balance Board
