# Supplementary material for: Dynamic balance and instrumented gait variables are independent predictors of falls following stroke
Source: J Neuroeng Rehabil. 2019 Jan 7;16:3. doi: 10.1186/s12984-018-0478-4 (PMC6322221; doi:10.1186/s12984-018-0478-4)
Supplement: Supplementary file 1 — (.pdf) Between-group differences and regression analyses with removal of those requiring physical assistance and/or gait aids (n = 57) (PDF 77 kb) [file 12984_2018_478_MOESM1_ESM.pdf]

Additional File 1. Between-group differences and regression analyses with removal of those requiring physical assistance and/or gait aids (n=57)

	**Non-fallers** (n=43) Mean±SD	**Fallers** (n=14) Mean±SD	**Between-group**[a] *P*-value	**25th – 75th quartiles**	**Regression adjusted for country and prior falls** IQR-OR	*P*-value	**Regression adjusted for country, prior falls and 6mWT** IQR-OR (95% CI)	*P*-value
Gait variables								
6mWT-comfortable, m/s (n=42:14)	0.93±0.28	0.75±0.24	0.077	0.69-1.04	2.30 (0.86-6.13)[b]	0.096	N/A	N/A
Kinect-fast walk, m/s (n=41:14)	1.10±0.27	0.91±0.30	0.066	0.87-1.27	2.53 (0.90-7.12)[b]	0.080	N/A	N/A
Stride length, m (n=41:14)	1.13±0.19	0.99±0.23	**0.039***	0.99-1.24	1.97 (0.87-4.42)[b]	0.102	1.13 (0.33-3.93)[b]	0.842
Cadence, steps/min (n=41:14)	116.62±15.56	107.32±21.65	0.127	106.05-125.88	1.72 (0.80-3.70)[b]	0.163	1.16 (0.44-3.04)[b]	0.760
Step width, m (n=41:14)	0.14±0.04	0.14±0.04	0.954	0.11-0.16	1.28 (0.46-3.58)[b]	0.633	1.65 (0.55-5.01)[b]	0.372
Step length asymmetry, ratio (n=41:14)[c]	1.10±0.10	1.09±0.07	0.728	1.05-1.12	1.08 (0.54-2.16)[b]	0.823	1.80 (0.67-*U*)[b]	0.246
Gait speed variability, m/s (n=41:14)[c]	0.19±0.07	0.14±0.04	**0.034***	0.12-0.19	2.57 (0.90-7.32)[b]	0.078	1.70 (0.47-6.21)[b]	0.419
ML pelvic displacement, cm (n=40:14)	6.60±1.13	5.26±1.13	**0.001***	5.42-7.04	**9.35 (2.14-40.86)[b]**	**0.003***	**8.54 (1.92-37.98)[b]**	**0.005***
Vertical pelvic displacement, cm (n=40:14)	3.82±1.15	3.07±0.88	**0.036***	2.73-4.21	2.42 (0.86-6.78)[b]	0.094	1.55 (0.41-5.86)[b]	0.516
Balance variables								
TUG - normal, s (n=43:14)[c]	12.34±4.01	16.18±7.51	**0.033***	9.79-15.59	2.58 (0.91-7.32)	0.075	2.40 (0.44-13.20)	0.313
TUG - dual task, s (n=41:13)[c]	16.44±11.18	19.44±9.81	0.112	11.23-19.03	1.29 (0.58-2.85)	0.531	1.31 (0.46-3.73)[b]	0.618
Step test - affected, taps/15s (n=43:14)	11.84±4.36	8.64±2.10	**0.014***	7.00-14.00	**5.47 (1.22-24.52)[b]**	**0.026***	5.02 (0.84-28.87)[b]	0.076
Step test - less affected, taps/15s (n=43:14)	10.56±4.34	6.14±2.48	**<0.001***	7.00-12.00	**6.19 (1.50-25.47)[b]**	**0.012***	**9.58 (1.55-59.28)[b]**	**0.015***
COP vel EO total, cm/s (n=42:13)[c]	1.31±0.46	1.55±1.10	0.663	1.07-1.44	1.01 (0.61-1.70)	0.955	1.00 (0.58-1.71)	0.986
COP vel EO ML, cm/s (n=42:13)[c]	0.54±0.17	0.71±0.58	0.766	0.42-0.66	1.44 (0.64-3.22)	0.377	1.42 (0.63-3.22)	0.402
COP vel EO AP, cm/s (n=42:13)[c]	1.07±0.44	1.23±0.83	0.890	0.83-1.18	1.06 (0.58-1.92)[b]	0.861	1.07 (0.57-2.01)[b]	0.832
COP vel EC total, cm/s (n=41:13)[c]	1.90±0.87	2.07±1.41	0.678	1.35-2.18	1.25 (0.61-2.57)[b]	0.540	1.26 (0.60-2.67)[b]	0.541
COP vel EC ML, cm/s (n=41:13)[c]	0.66±0.23	0.78±0.58	0.800	0.50-0.78	1.11 (0.55-2.21)	0.774	1.06 (0.52-2.18)	0.869
COP vel EC AP, cm/s (n=41:13)[c]	1.66±0.82	1.76±1.20	0.564	1.14-1.84	1.35 (0.67-2.71)[b]	0.407	1.35 (0.65-2.81)[b]	0.420

** Denotes a significance at P<0.05*

[a] Between-group differences assessed using Mann-Whitney U tests; [b] an inverse relationship with falls exists, therefore IQR-OR compares the odds of having more falls among individuals with predictor values at the 25th percentile with the odds of having more falls among individuals with predictor values at the 75th percentile; [c] log-transformed for regression analysis due to significant positive skew.

Abbreviations: 6mWT = 6-metre walk test; AP = anteroposterior; COP = centre of pressure; EC = eyes closed; EO = eyes open; IQR = interquartile range; ML = mediolateral; OR = odds ratio; TUG = timed up and go; U = unable to calculate
